# Accurate quantification of creatinine in serum by coupling a measurement standard to extractive electrospray ionization mass spectrometry

**DOI:** 10.1038/srep19283

**Published:** 2016-01-13

**Authors:** Keke Huang, Ming Li, Hongmei Li, Mengwan Li, You Jiang, Xiang Fang

**Affiliations:** 1 State Key Laboratory of Inorganic Synthesis and Preparative Chemistry, College of Chemistry, Jilin University, Changchun, 130012, P. R. China; 2 Chemistry Department, National Institute of Metrology, Beijing, 100013, P. R. China

## Abstract

Ambient ionization (AI) techniques have been widely used in chemistry, medicine, material science, environmental science, forensic science. AI takes advantage of direct desorption/ionization of chemicals in raw samples under ambient environmental conditions with minimal or no sample preparation. However, its quantitative accuracy is restricted by matrix effects during the ionization process. To improve the quantitative accuracy of AI, a matrix reference material, which is a particular form of measurement standard, was coupled to an AI technique in this study. Consequently the analyte concentration in a complex matrix can be easily quantified with high accuracy. As a demonstration, this novel method was applied for the accurate quantification of creatinine in serum by using extractive electrospray ionization (EESI) mass spectrometry. Over the concentration range investigated (0.166 ~ 1.617 μg/mL), a calibration curve was obtained with a satisfactory linearity (R^2^ = 0.994), and acceptable relative standard deviations (RSD) of 4.6 ~ 8.0% (n = 6). Finally, the creatinine concentration value of a serum sample was determined to be 36.18 ± 1.08 μg/mL, which is in excellent agreement with the certified value of 35.16 ± 0.39 μg/mL.

Renal failure (known as kidney failure) has received significant attention since even moderate reduction in kidney function is associated with high rate of mortality^1^. Early diagnosis of kidney disease, followed by appropriate medical treatments, can prevent or postpone kidney failure. Creatinine, a byproduct of muscle metabolism, is removed from the blood chiefly by the kidneys. The serum creatinine level is the most widely used and commonly accepted measure of renal function in clinical medicine^2^,^3^. Many techniques including electrochemical sensor^4^, Raman spectroscopy^5^, spectrophotometry^6^,^7^, capillary electrophoresis^8^,^9^, high performance liquid chromatography^10^, are available for the determination of creatinine in serum. Alternatively, hyphenated mass spectrometric methods including gas chromatography-mass spectrometry (GC/MS)^11^,^12^, liquid chromatography-mass spectrometry (LC/MS)^12^,^13^,^14^,^15^ have been developed for the accurate quantification of creatinine in serum. Among these techniques, liquid chromatography-isotope dilution mass spectrometry (LC-IDMS) was recommended as the reference technique by National Kidney Disease Education Program^16^. However, traditional MS method suffers from the need of tedious, time- and cost-consuming sample pretreatment steps (derivatization, extraction, chromatographic separation, etc.) before complex raw samples are analysed. Recently introduced ambient ionization techniques, including desorption electrospray ionization (DESI)^17^,^18^,^19^, direct analysis in real time (DART)^20^, desorption atmospheric pressure chemical ionization (DAPCI)^21^, dielectric barrier discharge ionization (DBDI)^22^, low temperature plasma probe (LTP)^23^, easy ambient sonic-spray ionization (EASI)^24^, and extractive electrospray ionization (EESI)^25^,^26^,^27^,^28^,^29^,^30^, allow direct desorption/ionization of chemicals in raw samples under ambient conditions. Ambient MS methods require no or minimal sample preparation and tolerate chemical contamination of an ion source. It is notable that DESI was adopted for the determination of urine creatinine level^19^. Nonetheless, current obstacle on the quantitative analysis of ambient mass spectrometry is the matrix effect^31^, especially in the case that samples have high complex matrix and high quantitative accuracy is required^32^. For example, the signal of protonated analytes will dramatically decrease in the case: i) the proton affinities (PA) of the species in matrix are larger than PA of the analyte, ii) alkali-metals (e.g. Na^+^, K^+^) in the matrix cause the formation of alkali-metal adducts of analyte. Therefore, a standard addition method or/and isotope dilution MS were coupled to ambient ionization technique to improve the analytical accuracy^33^,^34^. In this case, samples were prepared by spiking a series of stock solutions before being analyzed. For each sample analysis, at least five spiked samples had to be prepared, and consequently at least five MS analysis had to be performed^33^,^34^. It is still tedious, time- and cost-consuming to prepare and to analyse the spiked samples, especially for the high throughput analysis.

In this study, a simple, rapid ambient MS method was developed for accurate analysis of creatinine in serum by coupling a measurement standard to EESI.

## Results

### EESI-MS spectra of creatinine

**Figure 1:**
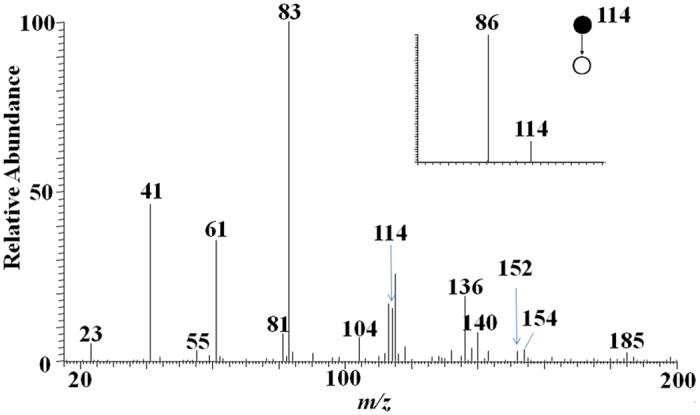
A typical EESI mass spectrum of a serum sample with 50 times dilution (inset shows an MS^2^ spectrum of *m/z* 114).

To optimize the parameters of the instrument, a creatinine standard solution (0.1 μg/mL) was introduced into EESI source and a typical mass spectrum of creatinine was recorded (see Figure S1). The signal at *m/z* 114 corresponds to protonated creatinine molecules. The product ion spectrum of the mass selected ions of *m/z* 114 (inset of Figure S1) generated ionic fragments of *m/z* 86 by the neutral loss of CO^35^. A serum sample (GBW 09170) diluted 50 times with water was directly analysed by EESI-MS, and the corresponding mass spectrum was recorded (see Fig. 1). The signal at *m/z* 114 corresponds to protonated creatinine molecules, which was confirmed by tandem mass spectrum (inset of Fig. 1). The signals at *m/z* 61, 83, 136 correspond to protonated urea, sodiated urea, and sodiated creatinine, respectively. Actually, sodium ion adducts are very common in MS^36^, and sodiated creatinine was also observed in ambient ionization mass spectrometry such as DESI^19^, EESI^30^, and contactless atmospheric pressure ionization^37^.

### Demonstration of the matrix effect

A series of creatinine standard solutions prepared with pure water were used to establish a calibration curve (see Figure S2). Afterwards, 50 fold dilution of GBW09171 was analysed by using EESI-MS. The creatinine level in the original serum sample was determined to be 13.96 ± 0.42 μg/mL, which is significantly lower than the certified value of 35.16 ± 0.39 μg/mL^15^. This is because matrix effect still exists during the ionization process even the serum sample was diluted 50 times with water. In addition, distribution of ion current between protonated creatinine (*m/z* 114) and sodiated creatinine (*m/z* 136) can also be a source of error.

### Establishment of a calibration curve with a matrix reference material

**Figure 2:**
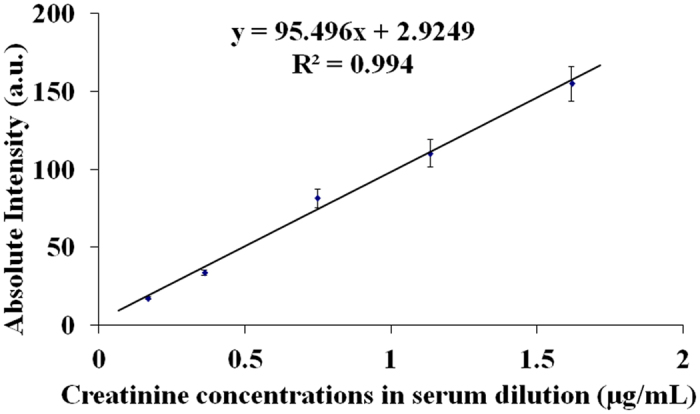
Calibration curve for determination of creatinine in serum with a transition ion pair *m/z* 114-86 as quantitative ions (Error bars designate the standard deviation, n = 6).

For reliable, accurate determination of creatinine level in serum sample, a matrix reference material, which is a particular form of measurement standard, was coupled to EESI technique. GBW09170 was chosen for constructing a calibration curve as it has a lower creatinine level. Five standard solutions, prepared with the matrix reference material, were analysed by using EESI-MS in MS^2^ mode. The intensity of quantitative ions of *m/z* 86, derived from the parent ions of *m/z* 114, was plotted as a function of creatinine concentration (see Fig. 2). Over the concentration range investigated (0.166 ~ 1.617 μg/mL), a calibration curve was obtained with a satisfactory linearity (R^2^ = 0.994), and acceptable relative standard deviations (RSD) of 4.6 ~ 8.0% (n = 6). The regression equation of the calibration curve was given as y = 95.5x + 2.93. The limit of quantification (S/N = 10) was calculated to be 0.03 μg/mL (i.e. 1.5 μg/mL in original serum sample). So the method reported here can be adopted for analysing the serum samples with creatinine level ranging 1.5 ~ 80.85 μg/mL, which is a suitable range for clinical application^38^. To assess the inter-day variation of the response, five calibration curves were established in five consecutive days with the same series of standard solutions (see Figure S3). The relative standard deviation of five slopes was calculated to be 0.49%, however it is suggested that a calibration curve should be re-established each time the EESI source is re-assembled. Here, the standard solutions were prepared with a matrix reference material, which has similar chemical characteristics to the samples being tested^15^. Therefore, this calibration curve can be used to accurately interpolate the creatinine level in serum samples.

### Quantification of creatinine in serum samples

A serum sample (GBW 09171) diluted 50 times with water was directly analysed by using EESI. The signal intensity in MS^2^ mode was recorded and the creatinine level in the original sample was calculated to be 36.18 ± 1.08 μg/mL. This value is in excellent agreement with the certified value of 35.16 ± 0.39 μg/mL, which was confirmed by an international comparison^15^. To assess the inter-assays variation, the same sample was analysed after one week, and the result was calculated to be 35.83 ± 1.01 μg/mL. The relative deviation between the results obtained from two different days is less than 0.5%. To further confirm the accuracy of this novel method, four serum samples were quantified via the proposed method and a reference method (i.e. LC-IDMS) developed previously in our lab^15^. The relative deviations of the results from these two methods were calculated to be 0.99–3.26%, as listed in Table 1.

## Discussion

Matrix effects are a current obstacle on the quantitative analysis of ambient mass spectrometry because of ion suppression, and distribution of ion current between protonated and sodiated molecules. To resolve this problem, a matrix reference material, which is considered as a “micro-ruler” to check the accuracy of tests and analytical procedures, was coupled to EESI technique. As a demonstration, this method was applied for the accurate quantification of creatinine in serum. The results show that the accurate analyte concentration in a complex matrix can be easily obtained with only one analysis for each sample by coupling a measurement standard to EESI.

## Methods

### Instrumentation and Working Conditions

**Figure 3:**
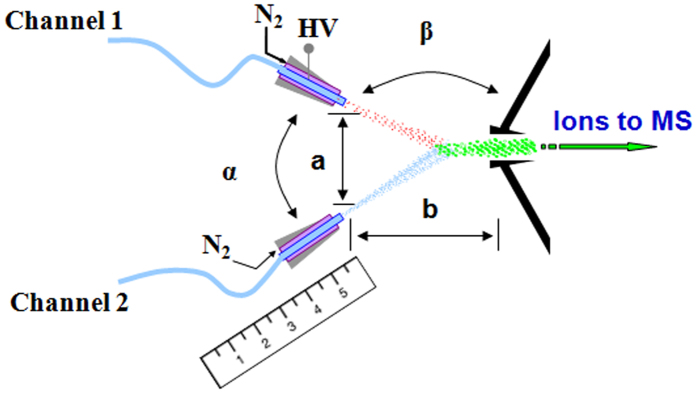
Schematic of the EESI source coupling with a measurement standard.

All the experiments were performed using a Thermo Finnigan LTQ mass spectrometer (San Jose, CA, USA) equipped with a homemade EESI source, which is described elsewhere^24^,^25^,^26^,^27^,^28^,^29^. A schematic of EESI source is shown in Fig. 3. A methanol/water/formic acid (v/v/v, 50:50:0.1) solution was injected into channel 1 using a syringe pump with a rate of 5 μL/min and nebulized with a sheath gas (N_2_). A high voltage of 4 kV was applied to channel 1 to generate the primary ions. Samples or standard solutions were injected into channel 2 using another syringe pump with a rate of 5 μL/min and also nebulized with a sheath gas (N_2_). The neutral analytes were ionized when intersecting with the primary ions. The angle (α) formed between the channel 1 and the channel 2 was 60°. The angle (β) formed between the channel 1 and the MS inlet was 150°. The distance (a) between the emitters of these two channels was 2 mm. The distance (b) between the EESI source and the MS inlet was 2.5 cm. The temperature of the ion entrance capillary was maintained at 390 °C during the experiment. For tandem mass spectrometry, the precursor ions of interest were isolated with a window width of 1.5 mass/charge units (full-width) and helium was used as a collisional gas. MS^2^ experiments were performed with an activation time of 40 ms and collision energy of 24% (arbitrary unit). All the mass spectra were recorded using Xcalibur^®^ software. The mean of the mass spectra accumulated for 1.5 min was recorded as one measurement result. An average of 6 measurements for one sample was used as the final result.

### Statement

All experimental protocols were approved by the ethics committee of National Institute of Metrology, China and adhered to the tenets of the Declaration of Helsinki. Additionally, the benefits and risks of this study were clearly presented to the volunteers, and thereafter the written consent was obtained if they agreed to join the study.

### Samples Preparation

Methanol (HPLC grade), and formic acid were purchased from Chinese Chemical Reagent Co., Ltd. (Shanghai, China). Ultrapure water (18.2 MΩ·cm^−1^) was prepared with Milli-Q Direct water purification system (Millipore, USA). Pure creatinine (SRM914a) with a purity of 99.7% was purchased from national institute of standards and technology (NIST), USA. Serum matrix reference materials GBW 09170 and GBW 09171 with certified creatinine values of 8.30 ± 0.10 μg/mL and 35.16 ± 0.39 μg/mL, were developed in our lab (i.e. national institute of metrology, China). Four serum samples, collected from volunteers including two healthy persons and two patients with kidney disease, were provided by the academy of military medical sciences (Beijing, China). All the chemicals were used directly without any further treatment.

## Additional Information

**How to cite this article**: Huang, K. *et al.* Accurate quantification of creatinine in serum by coupling a measurement standard to extractive electrospray ionization mass spectrometry. *Sci. Rep.* **6**, 19283; doi: 10.1038/srep19283 (2016).

## Supplementary Material

Supplementary Information

## Figures and Tables

**Table 1:** Comparison of creatinine level in serum by using LC-IDMS and EESI-MS.

Sample number	conc. by LC-IDMS (μg/mL)	conc. by EESI-MS (μg/mL)	Relative deviation (%)
1	7.67	7.42	3.26
2	9.03	8.85	2.00
3	22.52	23.19	2.98
4	33.34	32.01	0.99
