# Supplementary material for: Accurate quantification of creatinine in serum by coupling a measurement standard to extractive electrospray ionization mass spectrometry
Source: Sci Rep. 2016 Jan 13;6:19283. doi: 10.1038/srep19283 (PMC4725369; doi:10.1038/srep19283)
Supplement: Supplementary Information [file srep19283-s1.pdf]

Accurate quantification of creatinine in serum by coupling a measurement standard to extractive electrospray ionization mass spectrometry

Keke Huang[1], Ming Li[2,*], Hongmei Li[2], Mengwan Li[2], You Jiang[2], Xiang Fang[2]

[1]State Key Laboratory of Inorganic Synthesis and Preparative Chemistry, College of Chemistry, Jilin University, Changchun, 130012, P. R. China

[2]Chemistry Department, National Institute of Metrology, Beijing, 100013, P. R. China

* Corresponding author. e-mail: mingutah@hotmail.com; Tel: 86-10-64526792

Supplementary Information

Results

EESI-MS spectra of creatinine.

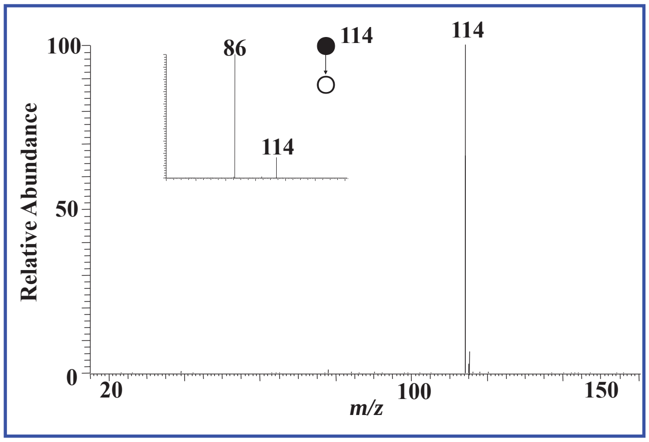

Figure S1 A typical EESI mass spectrum of a creatinine standard solution (0.1 μg/mL); inset shows an MS^2 spectrum of *m/z* 114

Demonstration of the matrix effect.

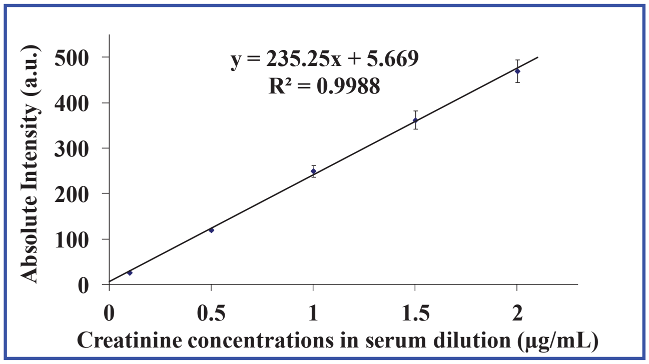

Figure S2 A calibration curve constructed with pure standard solutions (Error bars designate the standard deviation, n = 6)

Establishment of a calibration curve with a matrix reference material.

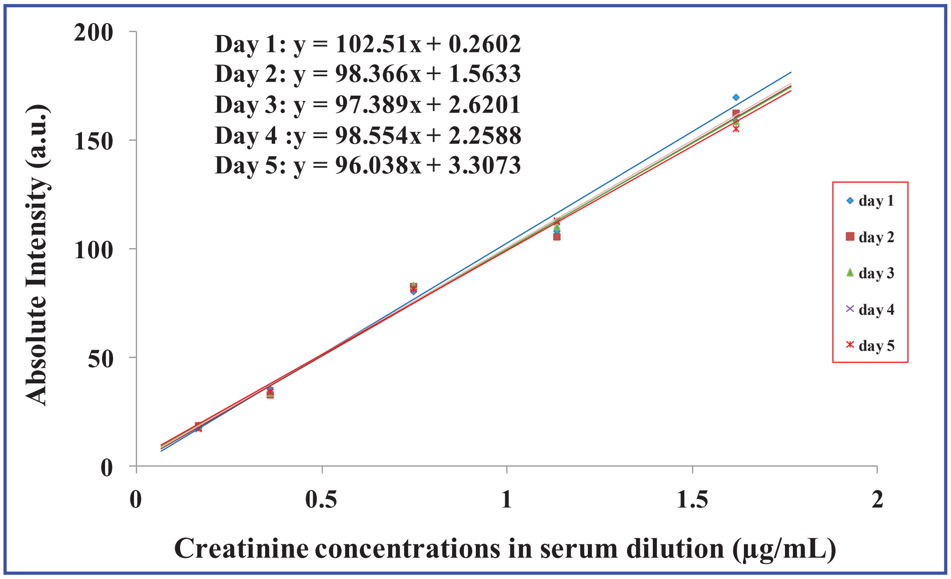

Figure S3 Calibration curves built up in five consecutive days

Preparation of standard solutions with matrix reference material. Five 10 μL of matrix reference material (GBW09170) were individually spiked with 0, 2, 4, 10, 15 μL of creatinine stock solution, followed by dilution with ultrapure water to a constant volume of 500 μL. The final concentrations of these five creatinine solutions were calculated to be 0.166, 0.359, 0.746, 1.133, 1.617 μg/mL, respectively.

Samples Preparation. A creatinine stock solution with a concentration of 48.35 μg/mL was prepared by dissolving 2.4249 mg of pure creatinine in 50 mL of ultrapure water. A creatinine standard solution with a concentration of 0.1 μg/mL was prepared by diluting the creatinine stock solution with ultrapure water.
